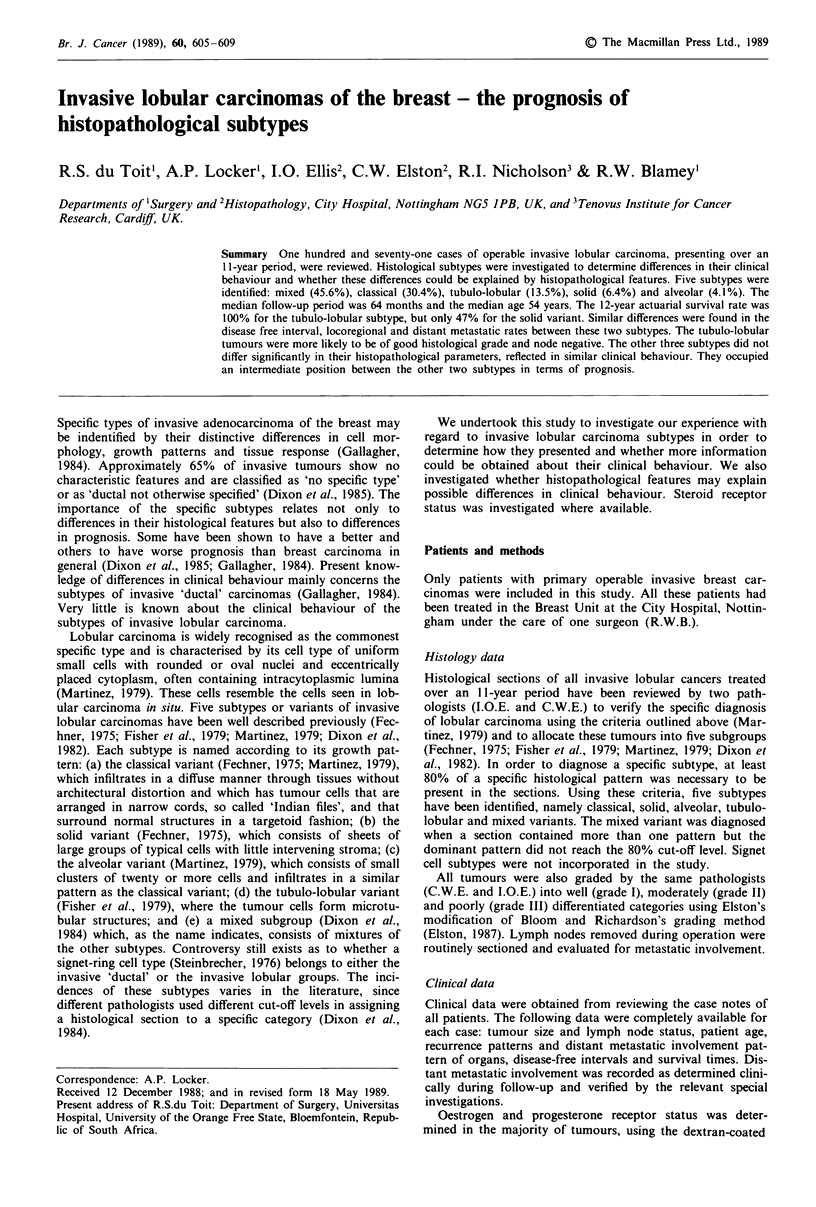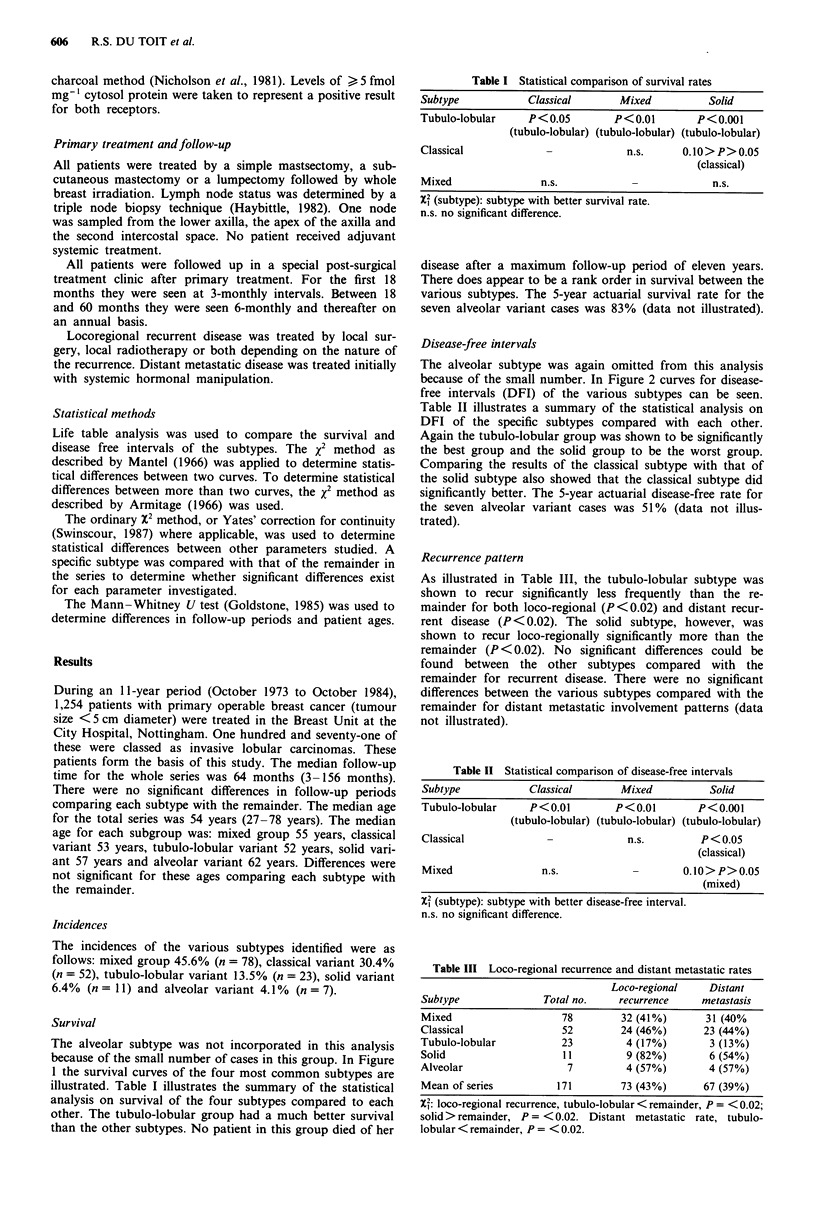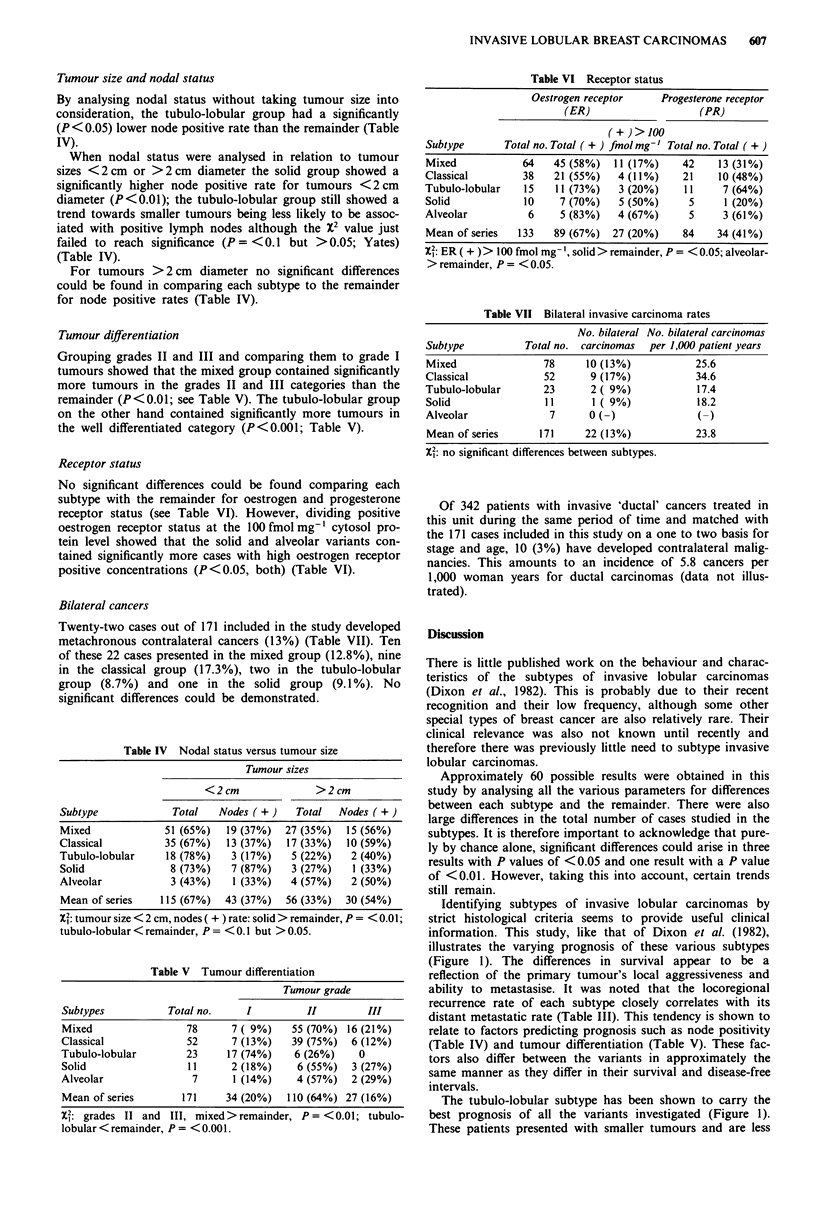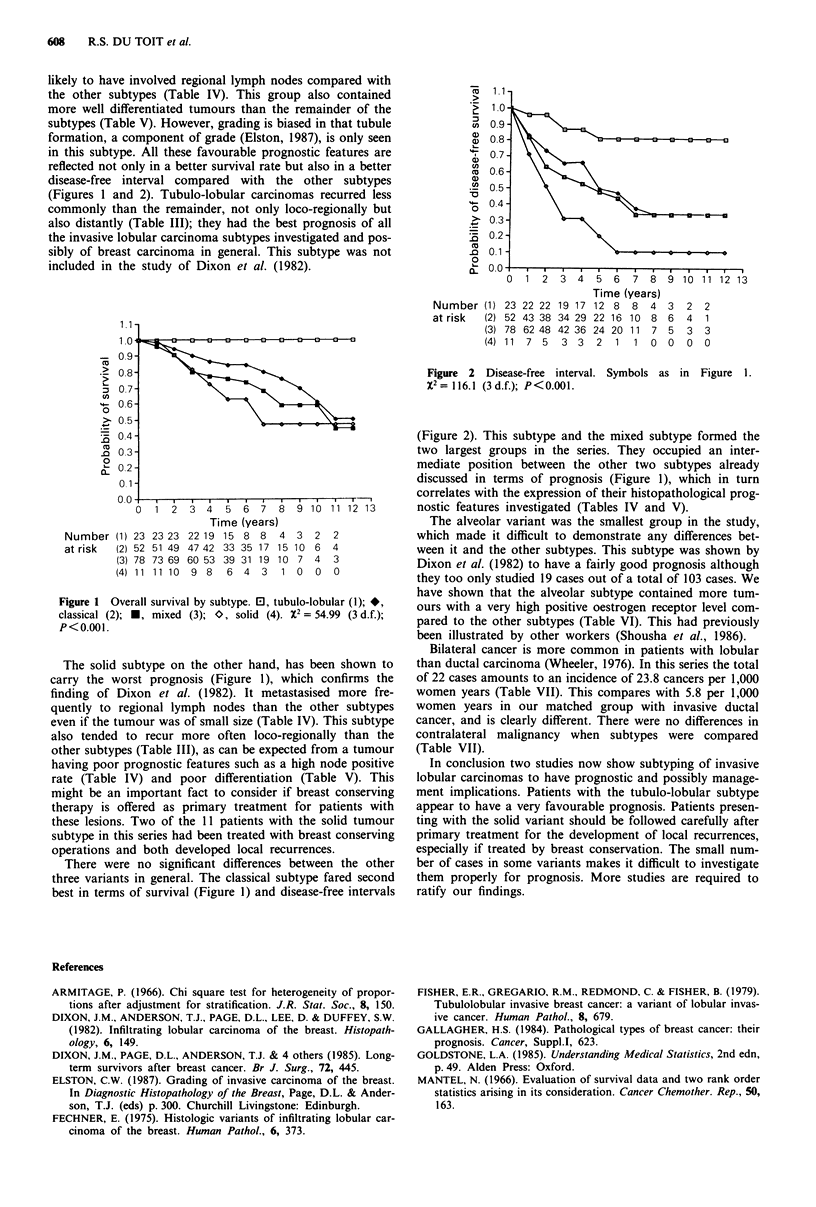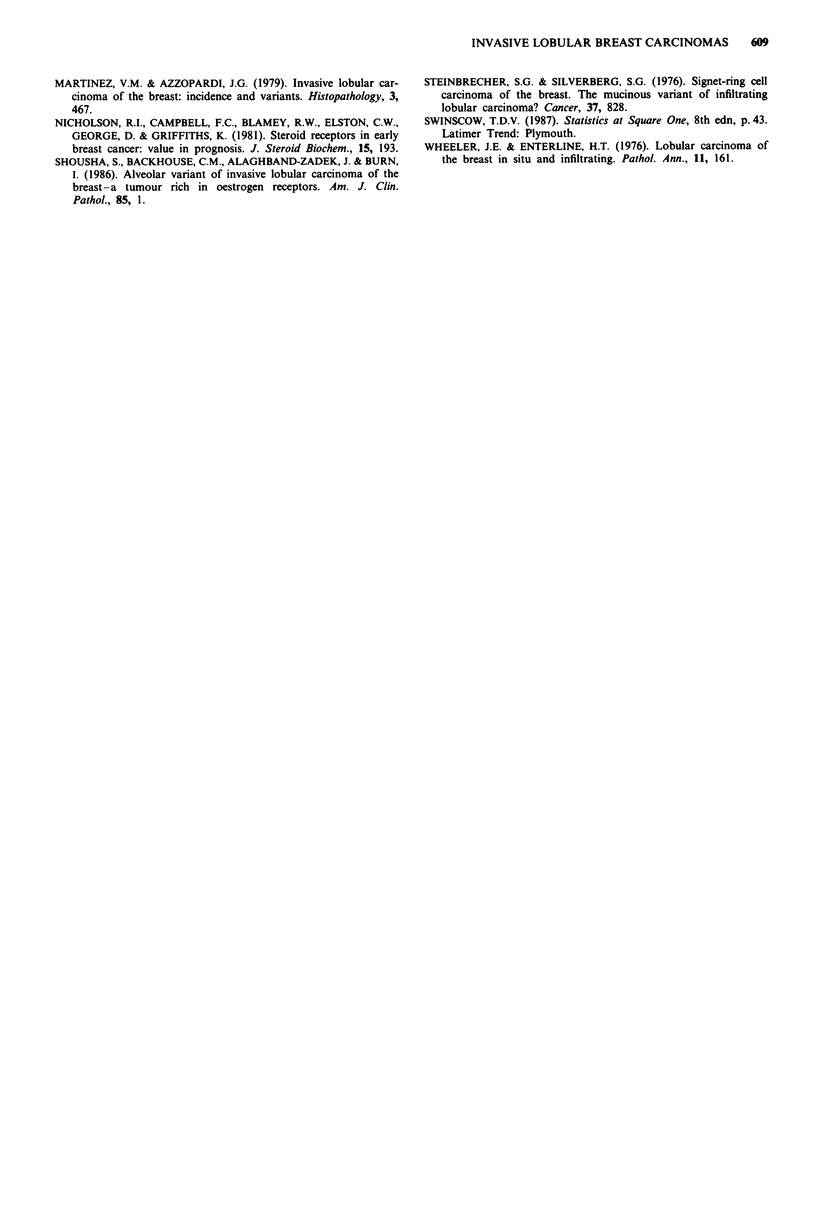# Invasive lobular carcinomas of the breast--the prognosis of histopathological subtypes.

**DOI:** 10.1038/bjc.1989.323

**Published:** 1989-10

**Authors:** R. S. du Toit, A. P. Locker, I. O. Ellis, C. W. Elston, R. I. Nicholson, R. W. Blamey

**Affiliations:** Department of Surgery, City Hospital, Nottingham, UK.

## Abstract

One hundred and seventy-one cases of operable invasive lobular carcinoma, presenting over an 11-year period, were reviewed. Histological subtypes were investigated to determine differences in their clinical behaviour and whether these differences could be explained by histopathological features. Five subtypes were identified: mixed (45.6%), classical (30.4%), tubulo-lobular (13.5%), solid (6.4%) and alveolar (4.1%). The median follow-up period was 64 months and the median age 54 years. The 12-year actuarial survival rate was 100% for the tubulo-lobular subtype, but only 47% for the solid variant. Similar differences were found in the disease free interval, locoregional and distant metastatic rates between these two subtypes. The tubulo-lobular tumours were more likely to be of good histological grade and node negative. The other three subtypes did not differ significantly in their histopathological parameters, reflected in similar clinical behaviour. They occupied an intermediate position between the other two subtypes in terms of prognosis.


Br. J. Cancer (1989), 60, 605-609 © The Macmillan Press Ltd., 1989

### Invasive lobular carcinomas of the breast – the prognosis of histopathological subtypes

R.S. du Toit[1], A.P. Locker[1], I.O. Ellis[2], C.W. Elston[2], R.I. Nicholson[3] & R.W. Blamey[1]

*Departments of [1]Surgery and [2]Histopathology, City Hospital, Nottingham NG5 1PB, UK, and [3]Tenovus Institute for Cancer Research, Cardiff, UK.*

**Summary** One hundred and seventy-one cases of operable invasive lobular carcinoma, presenting over an 11-year period, were reviewed. Histological subtypes were investigated to determine differences in their clinical behaviour and whether these differences could be explained by histopathological features. Five subtypes were identified: mixed (45.6%), classical (30.4%), tubulo-lobular (13.5%), solid (6.4%) and alveolar (4.1%). The median follow-up period was 64 months and the median age 54 years. The 12-year actuarial survival rate was 100% for the tubulo-lobular subtype, but only 47% for the solid variant. Similar differences were found in the disease free interval, locoregional and distant metastatic rates between these two subtypes. The tubulo-lobular tumours were more likely to be of good histological grade and node negative. The other three subtypes did not differ significantly in their histopathological parameters, reflected in similar clinical behaviour. They occupied an intermediate position between the other two subtypes in terms of prognosis.

Specific types of invasive adenocarcinoma of the breast may be indentified by their distinctive differences in cell morphology, growth patterns and tissue response (Gallagher, 1984). Approximately 65% of invasive tumours show no characteristic features and are classified as 'no specific type' or as 'ductal not otherwise specified' (Dixon *et al.*, 1985). The importance of the specific subtypes relates not only to differences in their histological features but also to differences in prognosis. Some have been shown to have a better and others to have worse prognosis than breast carcinoma in general (Dixon *et al.*, 1985; Gallagher, 1984). Present knowledge of differences in clinical behaviour mainly concerns the subtypes of invasive 'ductal' carcinomas (Gallagher, 1984). Very little is known about the clinical behaviour of the subtypes of invasive lobular carcinoma.

Lobular carcinoma is widely recognised as the commonest specific type and is characterised by its cell type of uniform small cells with rounded or oval nuclei and eccentrically placed cytoplasm, often containing intracytoplasmic lumina (Martinez, 1979). These cells resemble the cells seen in lobular carcinoma *in situ*. Five subtypes or variants of invasive lobular carcinomas have been well described previously (Fechner, 1975; Fisher *et al.*, 1979; Martinez, 1979; Dixon *et al.*, 1982). Each subtype is named according to its growth pattern: (a) the classical variant (Fechner, 1975; Martinez, 1979), which infiltrates in a diffuse manner through tissues without architectural distortion and which has tumour cells that are arranged in narrow cords, so called 'Indian files', and that surround normal structures in a targetoid fashion; (b) the solid variant (Fechner, 1975), which consists of sheets of large groups of typical cells with little intervening stroma; (c) the alveolar variant (Martinez, 1979), which consists of small clusters of twenty or more cells and infiltrates in a similar pattern as the classical variant; (d) the tubulo-lobular variant (Fisher *et al.*, 1979), where the tumour cells form microtubular structures; and (e) a mixed subgroup (Dixon *et al.*, 1984) which, as the name indicates, consists of mixtures of the other subtypes. Controversy still exists as to whether a signet-ring cell type (Steinbrecher, 1976) belongs to either the invasive 'ductal' or the invasive lobular groups. The incidences of these subtypes varies in the literature, since different pathologists used different cut-off levels in assigning a histological section to a specific category (Dixon *et al.*, 1984).

Correspondence: A.P. Locker.
Received 12 December 1988; and in revised form 18 May 1989.
Present address of R.S.du Toit: Department of Surgery, Universitas Hospital, University of the Orange Free State, Bloemfontein, Republic of South Africa.

We undertook this study to investigate our experience with regard to invasive lobular carcinoma subtypes in order to determine how they presented and whether more information could be obtained about their clinical behaviour. We also investigated whether histopathological features may explain possible differences in clinical behaviour. Steroid receptor status was investigated where available.

### Patients and methods

Only patients with primary operable invasive breast carcinomas were included in this study. All these patients had been treated in the Breast Unit at the City Hospital, Nottingham under the care of one surgeon (R.W.B.).

### *Histology data*

Histological sections of all invasive lobular cancers treated over an 11-year period have been reviewed by two pathologists (I.O.E. and C.W.E.) to verify the specific diagnosis of lobular carcinoma using the criteria outlined above (Martinez, 1979) and to allocate these tumours into five subgroups (Fechner, 1975; Fisher *et al.*, 1979; Martinez, 1979; Dixon *et al.*, 1982). In order to diagnose a specific subtype, at least 80% of a specific histological pattern was necessary to be present in the sections. Using these criteria, five subtypes have been identified, namely classical, solid, alveolar, tubulo-lobular and mixed variants. The mixed variant was diagnosed when a section contained more than one pattern but the dominant pattern did not reach the 80% cut-off level. Signet cell subtypes were not incorporated in the study.

All tumours were also graded by the same pathologists (C.W.E. and I.O.E.) into well (grade I), moderately (grade II) and poorly (grade III) differentiated categories using Elston's modification of Bloom and Richardson's grading method (Elston, 1987). Lymph nodes removed during operation were routinely sectioned and evaluated for metastatic involvement.

### *Clinical data*

Clinical data were obtained from reviewing the case notes of all patients. The following data were completely available for each case: tumour size and lymph node status, patient age, recurrence patterns and distant metastatic involvement pattern of organs, disease-free intervals and survival times. Distant metastatic involvement was recorded as determined clinically during follow-up and verified by the relevant special investigations.

Oestrogen and progesterone receptor status was determined in the majority of tumours, using the dextran-coated charcoal method (Nicholson *et al.*, 1981). Levels of $\geqslant 5$ fmol $mg^{-1}$ cytosol protein were taken to represent a positive result for both receptors.

*Primary treatment and follow-up*

All patients were treated by a simple mastsectomy, a subcutaneous mastectomy or a lumpectomy followed by whole breast irradiation. Lymph node status was determined by a triple node biopsy technique (Haybittle, 1982). One node was sampled from the lower axilla, the apex of the axilla and the second intercostal space. No patient received adjuvant systemic treatment.

All patients were followed up in a special post-surgical treatment clinic after primary treatment. For the first 18 months they were seen at 3-monthly intervals. Between 18 and 60 months they were seen 6-monthly and thereafter on an annual basis.

Locoregional recurrent disease was treated by local surgery, local radiotherapy or both depending on the nature of the recurrence. Distant metastatic disease was treated initially with systemic hormonal manipulation.

*Statistical methods*

Life table analysis was used to compare the survival and disease free intervals of the subtypes. The $\chi^2$ method as described by Mantel (1966) was applied to determine statistical differences between two curves. To determine statistical differences between more than two curves, the $\chi^2$ method as described by Armitage (1966) was used.

The ordinary $\chi^2$ method, or Yates' correction for continuity (Swinscour, 1987) where applicable, was used to determine statistical differences between other parameters studied. A specific subtype was compared with that of the remainder in the series to determine whether significant differences exist for each parameter investigated.

The Mann–Whitney $U$ test (Goldstone, 1985) was used to determine differences in follow-up periods and patient ages.

### Results

During an 11-year period (October 1973 to October 1984), 1,254 patients with primary operable breast cancer (tumour size $<5$ cm diameter) were treated in the Breast Unit at the City Hospital, Nottingham. One hundred and seventy-one of these were classed as invasive lobular carcinomas. These patients form the basis of this study. The median follow-up time for the whole series was 64 months (3–156 months). There were no significant differences in follow-up periods comparing each subtype with the remainder. The median age for the total series was 54 years (27–78 years). The median age for each subgroup was: mixed group 55 years, classical variant 53 years, tubulo-lobular variant 52 years, solid variant 57 years and alveolar variant 62 years. Differences were not significant for these ages comparing each subtype with the remainder.

*Incidences*

The incidences of the various subtypes identified were as follows: mixed group 45.6% ($n=78$), classical variant 30.4% ($n=52$), tubulo-lobular variant 13.5% ($n=23$), solid variant 6.4% ($n=11$) and alveolar variant 4.1% ($n=7$).

*Survival*

The alveolar subtype was not incorporated in this analysis because of the small number of cases in this group. In Figure 1 the survival curves of the four most common subtypes are illustrated. Table I illustrates the summary of the statistical analysis on survival of the four subtypes compared to each other. The tubulo-lobular group had a much better survival than the other subtypes. No patient in this group died of her disease after a maximum follow-up period of eleven years. There does appear to be a rank order in survival between the various subtypes. The 5-year actuarial survival rate for the seven alveolar variant cases was 83% (data not illustrated).

**Table I** Statistical comparison of survival rates

| *Subtype* | *Classical* | *Mixed* | *Solid* |
|---|---|---|---|
| Tubulo-lobular | $P<0.05$ (tubulo-lobular) | $P<0.01$ (tubulo-lobular) | $P<0.001$ (tubulo-lobular) |
| Classical | – | n.s. | $0.10>P>0.05$ (classical) |
| Mixed | n.s. | – | n.s. |

$\chi^2_1$ (subtype): subtype with better survival rate.
n.s. no significant difference.

*Disease-free intervals*

The alveolar subtype was again omitted from this analysis because of the small number. In Figure 2 curves for disease-free intervals (DFI) of the various subtypes can be seen. Table II illustrates a summary of the statistical analysis on DFI of the specific subtypes compared with each other. Again the tubulo-lobular group was shown to be significantly the best group and the solid group to be the worst group. Comparing the results of the classical subtype with that of the solid subtype also showed that the classical subtype did significantly better. The 5-year actuarial disease-free rate for the seven alveolar variant cases was 51% (data not illustrated).

*Recurrence pattern*

As illustrated in Table III, the tubulo-lobular subtype was shown to recur significantly less frequently than the remainder for both loco-regional ($P<0.02$) and distant recurrent disease ($P<0.02$). The solid subtype, however, was shown to recur loco-regionally significantly more than the remainder ($P<0.02$). No significant differences could be found between the other subtypes compared with the remainder for recurrent disease. There were no significant differences between the various subtypes compared with the remainder for distant metastatic involvement patterns (data not illustrated).

**Table II** Statistical comparison of disease-free intervals

| *Subtype* | *Classical* | *Mixed* | *Solid* |
|---|---|---|---|
| Tubulo-lobular | $P<0.01$ (tubulo-lobular) | $P<0.01$ (tubulo-lobular) | $P<0.001$ (tubulo-lobular) |
| Classical | – | n.s. | $P<0.05$ (classical) |
| Mixed | n.s. | – | $0.10>P>0.05$ (mixed) |

$\chi^2_1$ (subtype): subtype with better disease-free interval.
n.s. no significant difference.

**Table III** Loco-regional recurrence and distant metastatic rates

| *Subtype* | *Total no.* | *Loco-regional recurrence* | *Distant metastasis* |
|---|---|---|---|
| Mixed | 78 | 32 (41%) | 31 (40% |
| Classical | 52 | 24 (46%) | 23 (44%) |
| Tubulo-lobular | 23 | 4 (17%) | 3 (13%) |
| Solid | 11 | 9 (82%) | 6 (54%) |
| Alveolar | 7 | 4 (57%) | 4 (57%) |
| Mean of series | 171 | 73 (43%) | 67 (39%) |

$\chi^2_1$: loco-regional recurrence, tubulo-lobular < remainder, $P=<0.02$; solid > remainder, $P=<0.02$. Distant metastatic rate, tubulo-lobular < remainder, $P=<0.02$.

*Tumour size and nodal status*

By analysing nodal status without taking tumour size into consideration, the tubulo-lobular group had a significantly ($P<0.05$) lower node positive rate than the remainder (Table IV).

When nodal status were analysed in relation to tumour sizes <2 cm or >2 cm diameter the solid group showed a significantly higher node positive rate for tumours <2 cm diameter ($P<0.01$); the tubulo-lobular group still showed a trend towards smaller tumours being less likely to be associated with positive lymph nodes although the $\chi^2$ value just failed to reach significance ($P=<0.1$ but $>0.05$; Yates) (Table IV).

For tumours >2 cm diameter no significant differences could be found in comparing each subtype to the remainder for node positive rates (Table IV).

*Tumour differentiation*

Grouping grades II and III and comparing them to grade I tumours showed that the mixed group contained significantly more tumours in the grades II and III categories than the remainder ($P<0.01$; see Table V). The tubulo-lobular group on the other hand contained significantly more tumours in the well differentiated category ($P<0.001$; Table V).

*Receptor status*

No significant differences could be found comparing each subtype with the remainder for oestrogen and progesterone receptor status (see Table VI). However, dividing positive oestrogen receptor status at the 100 fmol mg$^{-1}$ cytosol protein level showed that the solid and alveolar variants contained significantly more cases with high oestrogen receptor positive concentrations ($P<0.05$, both) (Table VI).

*Bilateral cancers*

Twenty-two cases out of 171 included in the study developed metachronous contralateral cancers (13%) (Table VII). Ten of these 22 cases presented in the mixed group (12.8%), nine in the classical group (17.3%), two in the tubulo-lobular group (8.7%) and one in the solid group (9.1%). No significant differences could be demonstrated.

**Table IV** Nodal status versus tumour size

| | Tumour sizes | | | |
|---|---|---|---|---|
| | <2 cm | | >2 cm | |
| *Subtype* | *Total* | *Nodes ( + )* | *Total* | *Nodes ( + )* |
| Mixed | 51 (65%) | 19 (37%) | 27 (35%) | 15 (56%) |
| Classical | 35 (67%) | 13 (37%) | 17 (33%) | 10 (59%) |
| Tubulo-lobular | 18 (78%) | 3 (17%) | 5 (22%) | 2 (40%) |
| Solid | 8 (73%) | 7 (87%) | 3 (27%) | 1 (33%) |
| Alveolar | 3 (43%) | 1 (33%) | 4 (57%) | 2 (50%) |
| Mean of series | 115 (67%) | 43 (37%) | 56 (33%) | 30 (54%) |

$\chi^2_1$: tumour size <2 cm, nodes ( + ) rate: solid > remainder, $P=<0.01$; tubulo-lobular < remainder, $P=<0.1$ but $>0.05$.

**Table V** Tumour differentiation

| | | Tumour grade | | |
|---|---|---|---|---|
| *Subtypes* | *Total no.* | *I* | *II* | *III* |
| Mixed | 78 | 7 ( 9%) | 55 (70%) | 16 (21%) |
| Classical | 52 | 7 (13%) | 39 (75%) | 6 (12%) |
| Tubulo-lobular | 23 | 17 (74%) | 6 (26%) | 0 |
| Solid | 11 | 2 (18%) | 6 (55%) | 3 (27%) |
| Alveolar | 7 | 1 (14%) | 4 (57%) | 2 (29%) |
| Mean of series | 171 | 34 (20%) | 110 (64%) | 27 (16%) |

$\chi^2_1$: grades II and III, mixed > remainder, $P=<0.01$; tubulo-lobular < remainder, $P=<0.001$.

**Table VI** Receptor status

| | Oestrogen receptor (ER) | | | Progesterone receptor (PR) | |
|---|---|---|---|---|---|
| *Subtype* | *Total no.* | *Total ( + )* | *( + ) > 100 fmol mg$^{-1}$* | *Total no.* | *Total ( + )* |
| Mixed | 64 | 45 (58%) | 11 (17%) | 42 | 13 (31%) |
| Classical | 38 | 21 (55%) | 4 (11%) | 21 | 10 (48%) |
| Tubulo-lobular | 15 | 11 (73%) | 3 (20%) | 11 | 7 (64%) |
| Solid | 10 | 7 (70%) | 5 (50%) | 5 | 1 (20%) |
| Alveolar | 6 | 5 (83%) | 4 (67%) | 5 | 3 (61%) |
| Mean of series | 133 | 89 (67%) | 27 (20%) | 84 | 34 (41%) |

$\chi^2_1$: ER ( + ) > 100 fmol mg$^{-1}$, solid > remainder, $P=<0.05$; alveolar > remainder, $P=<0.05$.

**Table VII** Bilateral invasive carcinoma rates

| *Subtype* | *Total no.* | *No. bilateral carcinomas* | *No. bilateral carcinomas per 1,000 patient years* |
|---|---|---|---|
| Mixed | 78 | 10 (13%) | 25.6 |
| Classical | 52 | 9 (17%) | 34.6 |
| Tubulo-lobular | 23 | 2 ( 9%) | 17.4 |
| Solid | 11 | 1 ( 9%) | 18.2 |
| Alveolar | 7 | 0 (-) | (-) |
| Mean of series | 171 | 22 (13%) | 23.8 |

$\chi^2_1$: no significant differences between subtypes.

Of 342 patients with invasive 'ductal' cancers treated in this unit during the same period of time and matched with the 171 cases included in this study on a one to two basis for stage and age, 10 (3%) have developed contralateral malignancies. This amounts to an incidence of 5.8 cancers per 1,000 woman years for ductal carcinomas (data not illustrated).

### Discussion

There is little published work on the behaviour and characteristics of the subtypes of invasive lobular carcinomas (Dixon *et al.*, 1982). This is probably due to their recent recognition and their low frequency, although some other special types of breast cancer are also relatively rare. Their clinical relevance was also not known until recently and therefore there was previously little need to subtype invasive lobular carcinomas.

Approximately 60 possible results were obtained in this study by analysing all the various parameters for differences between each subtype and the remainder. There were also large differences in the total number of cases studied in the subtypes. It is therefore important to acknowledge that purely by chance alone, significant differences could arise in three results with $P$ values of $<0.05$ and one result with a $P$ value of $<0.01$. However, taking this into account, certain trends still remain.

Identifying subtypes of invasive lobular carcinomas by strict histological criteria seems to provide useful clinical information. This study, like that of Dixon *et al.* (1982), illustrates the varying prognosis of these various subtypes (Figure 1). The differences in survival appear to be a reflection of the primary tumour's local aggressiveness and ability to metastasise. It was noted that the locoregional recurrence rate of each subtype closely correlates with its distant metastatic rate (Table III). This tendency is shown to relate to factors predicting prognosis such as node positivity (Table IV) and tumour differentiation (Table V). These factors also differ between the variants in approximately the same manner as they differ in their survival and disease-free intervals.

The tubulo-lobular subtype has been shown to carry the best prognosis of all the variants investigated (Figure 1). These patients presented with smaller tumours and are less likely to have involved regional lymph nodes compared with the other subtypes (Table IV). This group also contained more well differentiated tumours than the remainder of the subtypes (Table V). However, grading is biased in that tubule formation, a component of grade (Elston, 1987), is only seen in this subtype. All these favourable prognostic features are reflected not only in a better survival rate but also in a better disease-free interval compared with the other subtypes (Figures 1 and 2). Tubulo-lobular carcinomas recurred less commonly than the remainder, not only loco-regionally but also distantly (Table III); they had the best prognosis of all the invasive lobular carcinoma subtypes investigated and possibly of breast carcinoma in general. This subtype was not included in the study of Dixon et al. (1982).

**Figure 1** Overall survival by subtype. □, tubulo-lobular (1); ◆, classical (2); ■, mixed (3); ◇, solid (4). $\chi^2 = 54.99$ (3 d.f.); $P<0.001$.

The solid subtype on the other hand, has been shown to carry the worst prognosis (Figure 1), which confirms the finding of Dixon et al. (1982). It metastasised more frequently to regional lymph nodes than the other subtypes even if the tumour was of small size (Table IV). This subtype also tended to recur more often loco-regionally than the other subtypes (Table III), as can be expected from a tumour having poor prognostic features such as a high node positive rate (Table IV) and poor differentiation (Table V). This might be an important fact to consider if breast conserving therapy is offered as primary treatment for patients with these lesions. Two of the 11 patients with the solid tumour subtype in this series had been treated with breast conserving operations and both developed local recurrences.

There were no significant differences between the other three variants in general. The classical subtype fared second best in terms of survival (Figure 1) and disease-free intervals

**Figure 2** Disease-free interval. Symbols as in Figure 1. $\chi^2 = 116.1$ (3 d.f.); $P<0.001$.

(Figure 2). This subtype and the mixed subtype formed the two largest groups in the series. They occupied an intermediate position between the other two subtypes already discussed in terms of prognosis (Figure 1), which in turn correlates with the expression of their histopathological prognostic features investigated (Tables IV and V).

The alveolar variant was the smallest group in the study, which made it difficult to demonstrate any differences between it and the other subtypes. This subtype was shown by Dixon et al. (1982) to have a fairly good prognosis although they too only studied 19 cases out of a total of 103 cases. We have shown that the alveolar subtype contained more tumours with a very high positive oestrogen receptor level compared to the other subtypes (Table VI). This had previously been illustrated by other workers (Shousha et al., 1986).

Bilateral cancer is more common in patients with lobular than ductal carcinoma (Wheeler, 1976). In this series the total of 22 cases amounts to an incidence of 23.8 cancers per 1,000 women years (Table VII). This compares with 5.8 per 1,000 women years in our matched group with invasive ductal cancer, and is clearly different. There were no differences in contralateral malignancy when subtypes were compared (Table VII).

In conclusion two studies now show subtyping of invasive lobular carcinomas to have prognostic and possibly management implications. Patients with the tubulo-lobular subtype appear to have a very favourable prognosis. Patients presenting with the solid variant should be followed carefully after primary treatment for the development of local recurrences, especially if treated by breast conservation. The small number of cases in some variants makes it difficult to investigate them properly for prognosis. More studies are required to ratify our findings.

### References

ARMITAGE, P. (1966). Chi square test for heterogeneity of proportions after adjustment for stratification. *J.R. Stat. Soc.*, **8**, 150.

DIXON, J.M., ANDERSON, T.J., PAGE, D.L., LEE, D. & DUFFEY, S.W. (1982). Infiltrating lobular carcinoma of the breast. *Histopathology*, **6**, 149.

DIXON, J.M., PAGE, D.L., ANDERSON, T.J. & 4 others (1985). Long-term survivors after breast cancer. *Br J. Surg.*, **72**, 445.

ELSTON, C.W. (1987). Grading of invasive carcinoma of the breast. In *Diagnostic Histopathology of the Breast*, Page, D.L. & Anderson, T.J. (eds) p. 300. Churchill Livingstone: Edinburgh.

FECHNER, E. (1975). Histologic variants of infiltrating lobular carcinoma of the breast. *Human Pathol.*, **6**, 373.

FISHER, E.R., GREGARIO, R.M., REDMOND, C. & FISHER, B. (1979). Tubulolobular invasive breast cancer: a variant of lobular invasive cancer. *Human Pathol.*, **8**, 679.

GALLAGHER, H.S. (1984). Pathological types of breast cancer: their prognosis. *Cancer*, Suppl.I, 623.

GOLDSTONE, L.A. (1985). *Understanding Medical Statistics*, 2nd edn, p. 49. Alden Press: Oxford.

MANTEL, N. (1966). Evaluation of survival data and two rank order statistics arising in its consideration. *Cancer Chemother. Rep.*, **50**, 163.

MARTINEZ, V.M. & AZZOPARDI, J.G. (1979). Invasive lobular carcinoma of the breast: incidence and variants. *Histopathology*, **3**, 467.

NICHOLSON, R.I., CAMPBELL, F.C., BLAMEY, R.W., ELSTON, C.W., GEORGE, D. & GRIFFITHS, K. (1981). Steroid receptors in early breast cancer: value in prognosis. *J. Steroid Biochem.*, **15**, 193.

SHOUSHA, S., BACKHOUSE, C.M., ALAGHBAND-ZADEK, J. & BURN, I. (1986). Alveolar variant of invasive lobular carcinoma of the breast–a tumour rich in oestrogen receptors. *Am. J. Clin. Pathol.*, **85**, 1.

STEINBRECHER, S.G. & SILVERBERG, S.G. (1976). Signet-ring cell carcinoma of the breast. The mucinous variant of infiltrating lobular carcinoma? *Cancer*, **37**, 828.

SWINSCOW, T.D.V. (1987). *Statistics at Square One*, 8th edn, p. 43. Latimer Trend: Plymouth.

WHEELER, J.E. & ENTERLINE, H.T. (1976). Lobular carcinoma of the breast in situ and infiltrating. *Pathol. Ann.*, **11**, 161.